# The Prevalence of Sexual Violence Perpetration in Sexual Minority Men: A Secondary Analysis of Systematic Review Data

**DOI:** 10.5964/ejop.6127

**Published:** 2022-11-30

**Authors:** RaeAnn E. Anderson, Sara K. Kuhn, Amanda M. Vitale, Alyssa M. Ciampaglia, Kristin E. Silver

**Affiliations:** 1 Department of Psychology, University of North Dakota, Grand Forks, ND, USA; 2 James J. Peters Veterans Affairs Medical Center, New York, NY, USA; 3 Icahn School of Medicine at Mount Sinai, New York, NY, USA; 4 Neuro-Oncology Department, Children’s Hospital of Philadelphia, Philadelphia, PA, USA; 5 Department of Neurology, Duke University, Durham, NC, USA; 6 Durham Veterans Affairs Medical Center, Durham, NC, USA; Edinburgh Napier University, Edinburgh, United Kingdom

**Keywords:** sexual minority men, sexual perpetration, campus sexual assault, measurement, prevalence

## Abstract

Prior literature illustrates that sexual minority people (e.g., bisexual, gay, queer) are at increased vulnerability for sexual violence victimization compared to heterosexual peers, including while in college. However, the study of sexual violence perpetration in sexual minority populations, much less specifically sexual minority college men, has been neglected. This article reviews the literature and presents a secondary data analysis of a systematic review on college men’s sexual perpetration rates and associated methodology. We also conducted analyses to summarize available literature regarding publishing dates, authors, and data inclusivity. Methods: We downloaded the dataset and associated materials from Mendeley.com’s data archive. Results: To our surprise, we could not analyze sexual perpetration prevalence rates in sexual minority men using the systematic review data due to absence of reported data across all 77 independent samples including over 5,500 male participants. We found no significant relationship between inclusion of sexual minority men and the use of measurement strategies specialized to assess sexual minority needs. We did find a positive relationship between recency of publication and the inclusion of sexual minority men, r(76) = .24, p = .03, and that most authors/co-authors were women (72%). Conclusions: Preventing perpetration is central to ending sexual violence; therefore, future research should include sexual minority people and use appropriate methodology in the investigation of sexual perpetration characteristics and patterns.

Sexual violence victimization, the experience of sexual contact without consent, is a significant public health problem. Sexual victimization increases vulnerability to a broad range of long-term physical and mental health issues including depression, eating disorders, chronic pain, and posttraumatic stress disorder (Dworkin et al., 2017), and for men there is a likelihood of: suicidal ideation, suicide attempts and/or self-harm; depression; heavy drinking or drug use and resultant organ failure; somatic symptoms; sexual dysfunction; STIs/HIV; and general poor health (Sampsel, 2016). Sexual minority students (those who identify as gay, queer, bisexual, or any other non-heterosexual sexual identity) are at increased vulnerability compared to their heterosexual peers (Eisenberg et al., 2021; Martin-Storey et al., 2018; Rothman et al., 2011). However, the literature predominantly focuses on a heteronormative model of cisgender heterosexual men assaulting cisgender heterosexual women (Anderson et al., 2021; Turchik et al., 2016). For example, heteromantic preferences can exclude SM people and others who may engage in non-monogamous sexual partnerships (Ison, 2019). Thus, sexual violence is yet another area in which sexual and gender minority (SGM) groups experience disparities in representation and health (Wallace & Santacruz, 2017; Westefeld et al., 2001). Additionally, homophobia and heteronormativity perpetuate the myth that sexual violence does not occur in LGBTQ+ communities (Ison, 2019).

Further, the study of sexual violence must also include the study of sexual perpetration, although this has been historically neglected (McKay et al., 2019). Sexual victimization and sexual perpetration are two halves of the same phenomenon—sexual violence. By neglecting the study of perpetration, we neglect the possibility of discovering key prevention strategies (Abbey, 2005). Thus, the goal of this study was to review the literature to ascertain what the prevalence rate of sexual perpetration is among sexual minority men (SMM) in college and the attendant research methodologies used to determine these prevalence rates. We chose to focus specifically on perpetration behavior, not to demonize or pathologize an already stigmatized and vulnerable group of people who are victims themselves (i.e., SMM), but to acknowledge the centrality of perpetration to true sexual violence prevention and address the neglect of this topic thus far in the literature. Our sample will be SMM in college in the United States and Canada as we are reviewing an available dataset comprising existing literature on perpetration behavior in this population. We also wanted to guard against introducing variance due to culture given findings from a recent meta-analysis revealing a wide range of prevalence rates within European countries (Krahé et al., 2014). Additionally, we will not sample the literature on known adult sex offenders (e.g., prison samples; registered sex offenders) as they are a very small percentage of those who perpetrate. Rates of sexual perpetration in college men is around 29% (Anderson et al., 2020) while registered sex offenders make up less than half a percent of the United States’ population (NCMEC, 2017). Furthermore, due to the prevalence of alcohol use and the sexual culture in colleges, students frequent locations and events that are at high risk for sexual violence (Fedina et al., 2018).

## Sexual Minority People’s Vulnerability for Sexual Victimization

A wealth of research has recently emerged which documents that SGM people (e.g., LGBTQ+ people, LGB and straight trans men and women, intersex and non-binary people, and gender non-conforming or gender-queer people who utilize “they/them” pronouns) are at increased vulnerability for experiencing sexual violence victimization (Hughes et al., 2010; Rothman et al., 2011; Walters et al., 2013). According to the CDC, cisgender gay and bisexual men are 1.9 and 2.3 times (respectively) more likely to experience rape and sexual violence than heterosexual men (Walters et al., 2013); among SMM college men, they are 3x more likely to experience rape (Anderson et al., 2017). Put another way, this suggests that 40.2 to 47.4% of SMM experience sexual victimization (Walters et al., 2013). Consistent with history, not only have SGM people’s experiences been neglected in this research, so have the experiences of other minoritized identities such as people of color (Griner et al., 2020). Yet, understanding the source of victimization (e.g., the behavior of the perpetrators) is fundamentally limited when solely examining experiences of those who have been victimized. Thus, comprehensive prevention for sexual minority groups requires examining the behavior of those who harm sexual minority people.

## Who Is Harming Sexual Minority People?

It might be useful to look towards related violence research on SGM populations for a clue as to the prevalence of sexual perpetration among SGM people. For example, a recent study on female-assigned-at-birth SGM people (e.g., transgender men, LGBTQ+ cisgender women) found only 2.6% reported intimate partner sexual violence perpetration (Messinger et al., 2021) while rates of reported victimization were much higher. Indeed, research on hate crimes suggest that approximately 20% of sexual minority adults reported experiencing a hate crime directed at their person or property due to their sexual identity (Burks et al., 2018; Herek, 2009). Consistent with minority stress theory and the hate crime data, lifetime discrimination has been associated with intimate partner violence (IPV) perpetration (Shorey et al., 2019), suggesting that the strain of stigma leads to internalized (e.g., anxiety, depression) as well as externalized (e.g., substance use, violence) behavior. Another IPV focused study of SMM found that 44.9% of SMM reported lifetime IPV victimization while 19.5% reported lifetime IPV perpetration (Miltz et al., 2019), raising the question of where this threat of sexual violence is coming from. Within the LGBTQ+ community, outside the community, or both, and under what circumstances?

An important methodology for answering this question is comparing rates of reported sexual violence perpetration in groups of men who differ by sexual identity. We conducted a thorough review of the literature and located only three relevant studies which surveyed community or college samples and assessed the history of sexual perpetration behavior and sexual identity. We excluded incarcerated samples as the environmental stressors and vulnerabilities differ greatly from community/college samples.

## Evidence From the Literature: Comparison of Self-Reported Perpetration Rates by Sexual Identity

Anderson et al. (2017) found no differences between sexual minority and heterosexual American college men in reported rates of sexual violence perpetration. In contrast, Krahé and Berger (2013) found that German college men, who were classified behaviorally as bisexual (rather than by self-identification), reported the highest rates of sexual perpetration, followed by behaviorally heterosexual men and behaviorally gay men. Walsh et al.’s (2021) results contrasted with those of both Anderson et al. (2017) and Krahé and Berger (2013), finding that heterosexual college men had the highest reported sexual violence perpetration rates. The methodological differences across these studies are one possible source of differences. The Anderson et al. (2017) and Walsh et al. (2021) studies recruited small samples of SMM. Krahé and Berger (2013) defined sexual orientation behaviorally, rather than by individual identity, as Anderson et al. (2017) and Walsh et al. (2021) had. All three studies used different measurement strategies to assess sexual violence perpetration; prior research has documented that the wide variation in sexual violence prevalence rates is tied to measurement issues (Anderson et al., 2021; Fedina et al., 2018; Peterson et al., 2011). This small body of research typifies the issues underscored by McKay et al. (2019) in their research on violence against LGBTQ people—there is a lack of standardized, comprehensive measures for violence in LGBTQ populations and a lack of attention towards perpetration research. Further research is needed on sexual perpetration that includes SMM and assesses sexual violence in ways that are inclusive of SMM’s unique experiences.

## Measurement and Methodology Issues in Sexual Violence

In general, men’s experiences of sexual violence victimization are under-researched (Davies, 2002). Until recently—and similar to current United Kingdom law (Weare, 2021)—most federal definitions of sexual violence in the United States excluded certain experiences that are more common for people with penises, such as being made to penetrate someone (Stemple & Meyer, 2014). Made to penetrate victimization may be particularly relevant to understanding bisexual men’s experiences, as some data suggests this behavior is perpetrated almost exclusively by heterosexual women (Anderson et al., 2020; Weare, 2018). Further, research suggests that currently available questionnaires, such as the iterations of the Sexual Experiences Survey, may be inherently gendered and contain some degree of heterosexist bias because the development of these questionnaires largely excluded SGM populations (Anderson & Delahanty, 2020; Anderson et al., 2021). Indeed, research from the IPV literature suggests there may be tactics of violence specific to the experiences of SGM people, such as threatening to “out” someone (i.e., reveal their sexual identity without their consent; Balsam & Szymanski, 2005; Dyar et al., 2021). Thus, research on sexual violence and SMM must use tools that encompass the range of sexual experience SGM may report, while simultaneously not stigmatizing or judging. The lack of appropriate measurement tools has been an ongoing challenge in decades of research on violence against SGM people (McKay et al., 2019).

## Current Study

Our literature review located three prior studies (not included in the systematic review) on the topic of SMM and sexual perpetration. Each demonstrated that measurement strategy may be obscuring the relationship between perpetration rates and sexual identity. The purpose of this study is to conduct a secondary data analysis of the most recent systematic review data (Anderson et al., 2021) on sexual perpetration in college men in the United States and Canada to examine how prevalence rates and associated measurement strategies may differ by sexual identity. Because the original systematic review did not focus on SMM data, we reanalyzed the data for this purpose consistent with calls in the literature to focus on perpetration and on measurement research, respectively (McKay et al., 2019).

**Research Question (RQ) 1**. We compared perpetration prevalence rates for SMM compared to heterosexual men. We did not make specific hypotheses given the mixed findings in the literature.
**H1**. We hypothesize that studies which included SMM (and did not exclude them from analyses) will be more likely to use modifications that would be more inclusive and more sensitive to known vulnerability factors for sexual minority people, given that over half the studies used modified measures. Such modifications include gender neutral wording, adding substance use items, and follow-up questions about the context of the assault given prior research on the potentially heterosexist nature of traditional questionnaires (Anderson & Delahanty, 2020; Koss et al., 2007).
**H2**. We will also explore whether studies that included SMM were more likely to use specific questionnaires or questionnaire types. We consider these analyses to be exploratory given the lack of existing data.
**H3**. Finally, given the paucity of relevant literature, we conducted analyses to characterize available literature. We hypothesize that there will be a relationship between year of publication and inclusion of SMM, with more recent publications being more likely to include SMM given the relatively recent increase in publications on sexual minority health (Coulter et al., 2014).
**RQ2**. We also characterized the discipline and gender of authors of relevant literature and whether the literature was inclusive of racial/ethnic minorities to identify potential targets for systematic efforts to increase equity and diversity in this research area.

## Method

### Participants

As reported in the original systematic review (Anderson et al., 2021), participants were 25,524 college men recruited across 78 independent samples that reported prevalence rates of sexual violence perpetration and were published between 2000–2017. This open access dataset is publicly available via Mendeley.com (Anderson, 2019, June 21). Participants were all men (100%), mostly white (76.8%) and heterosexual (97.5%). Zero studies in both the open access dataset, and the analytic sample in this paper, included gender minorities.

### Measures

Measurement strategy was coded in detail including the name of the questionnaire, modifications made to the questionnaire, and procedure of administration. In brief, at least 16 different questionnaires were reported across the 77 included studies; the most common questionnaire was the 1982 Sexual Experiences Survey (SES). Most studies used a version of the SES (78.2%) and modified the primary questionnaire in some way (61.1%).

### Procedures

For the purpose of this secondary data analysis, we focused on a subset of these 77 studies that included sexual minority men (SMM), *N* = 24. See Table A1 in the Appendix for a list of these 24 studies. These studies were re-analyzed for prevalence rates of perpetration in SMM and variation in measurement strategy that are sensitive to sexual and gender minority (SGM) samples. In the case of datasets which resulted in multiple publications, the original study included only the citation with the most relevant data. Thus, we reviewed the list of excluded studies for any included SMM data. Gender of study authors was determined via pronouns appearing on professional or personal websites and/or email communication with the authors.

## Results

### Analytic Plan

Chi square analyses (χ*^2^*) were used to examine the relationships between reported prevalence rates and indicators of sexual minority men (SMM) inclusion (see Table 1). The only exception was in the case of “date of publication” for which a Pearson correlation coefficient (*r*) was employed (see Table 1). Next we describe this strategy in relation to each research question (RQ) and hypothesis (H).

**Table 1:** Relationships Between Sexual Minority Men Participant Data Inclusion and Study Variables

Variable	Included SMM Data (*n* = 24, % or *M*)	Excluded SMM Data (*n* = 54, % or *M*)	Statistics
Perpetration Rates			
Average rate of any sexual violence	26.23%	28.91%	χ*^2^*(1, *N* = 78) = .06, *p* = .81
Rape perpetration prevalence rates	6.32%	6.67%	χ*^2^*(1, *N* = 43) = .003, *p* = .95
Verbal coercion rates	16.46%	20.88%	χ*^2^*(1, *N* = 49) = .20, *p* = .65
Study Characteristics			
Used any version of the SES	79.2%	72.2%	χ*^2^*(1, *N* = 20) = .42, *p* = .59
Primary questionnaire used for sexual perpetration was standardized	91.7%	92.6%	χ*^2^*(1, *N* = 6) = .02, *p* = 1.00
Date of publication	2012	2010	*r*(76) = .24, *p* = .03
Total percentage of racial minority participants	22.83%	23.35%	χ*^2^*(1, *N* = 74) = .002, *p* = .96
Questionnaire Modification			
Gender neutral wording	4.2%	7.4%	χ*^2^*(1, *N* = 73) = .29, *p* = 1.00
Verbal coercion item addition	8.3%	13.0%	χ*^2^*(1, *N* = 69) = .35, *p* = .71
Alcohol and substance use item addition	20.8%	22.2%	χ*^2^*(1, *N* = 61) = .02, *p* = 1.00
Altered definition of consent	—	—	—
Revision of instructions/response scale	29.2%	25.9%	χ*^2^*(1, *N* = 57) = .09, *p* = .79
Item removal	8.3%	9.3%	χ*^2^*(1, *N* = 71) = .02, *p* = 1.00
Item combination	4.2%	1.9%	χ*^2^*(1, *N* = 76) = .36, *p* = .52
Inclusion of follow-up items regarding assault context	8.3%	7.4%	χ*^2^*(1, *N* = 72) = .02, *p* = 1.00
Addition of sexual outcome items	12.5%	11.1%	χ*^2^*(1, *N* = 69) = .03, *p* = 1.00
Addition of items is unclear	12.5%	7.4%	χ*^2^*(1, *N* = 71) = .53, *p* = .67

**RQ 1**. Since we were unable to analyze perpetration prevalence rates by sexual identity due to lack of data, perpetration prevalence rates for studies that included SMM compared to studies which did not were examined through chi square analyses comparing the average rate of perpetration.
**H1**. To examine if studies that included and analyzed SMM data were more likely than those that did not to use sexual minority inclusive questionnaire modifications, we used chi square analyses to compare the following variables across the two groups (see Table 1–Questionnaire Modification): 1) the use of gender neutral wording (e.g., no male/female pronouns), 2) if an item for verbal coercion was added, 3) if an item for alcohol/substance use was added, 4) if the authors altered the definition of consent for the questionnaire, 5) if the instructions or response scales were revised, 6) if items were removed, 7) if items were combined, 8) if the authors added follow-up items to determine the context of the sexual assault such as setting or perpetrator characteristics, 9) if sexual outcome items (i.e., questions regarding specific sexual behaviors such as oral/anal penetration) were added, and 10) if it was unclear whether items were added or not.
**H2**. We utilized chi square analyses to determine if studies including and analyzing SMM data were more likely to use any version of the Sexual Experiences Survey (SES) or any standardized questionnaire (see Table 1–Study Characteristics).
**H3**. A Pearson correlation coefficient was employed to determine if there was a relationship between the year/date of study publication and the inclusion and analysis of SMM data (see Table 1–Study Characteristics).
**RQ2**. Chi square analyses were used to determine if the inclusion and analysis of SMM data was related to the inclusion of racial/ethnic minority participants (see Table 1–Study Characteristics). Author gender and discipline for studies including and analyzing SMM data were evaluated through a simple numerical count and related percentages.

### Research Question 1–Prevalence Rates

None of the included articles (*N* = 24), comprising 5,795 participants who identified as men, reported perpetration rates by sexual identity; therefore, our first research question could not be analyzed. Even in examining the exclusion lists for additional analyses of these samples, we were stymied by a lack of data. This lack of reporting may be due to small SMM participant sample sizes (less than 15% of total sample). Specifically, 21 of the 24 studies reporting SMM participant data were at least 90% heterosexual.

### Hypothesis 1 and 2–Measurement Strategy

There was no significant relationship between the inclusion of SMM participant data and the use of questionnaire modifications that would increase inclusivity and be more relevant to a sexual minority sample. Nor were studies including SMM more likely to use a specific questionnaire for perpetration; 20 (83.3%) of the 24 studies that reported collecting SMM participant data used a version of the SES.

### Hypothesis 3–Date of Publication

Statistical analysis revealed a significant relationship between studies that reported including data from SMM participants and the date of study publication (see Table 1). More recent studies (*N* = 24) reported including SMM data, with a mean publication year of 2012 and a modal year of 2017 (*n* = 6), compared to a mean year of 2010 and modal year of 2016 (*n* = 9) for all 77 articles analyzed.

### Research Question 2–Characterizing the Literature: Author Gender and Discipline

Finally, we decided to examine if inclusion of sexual minority people was related to inclusion in other ways. The inclusion of SMM participant data was not significantly related to the inclusion of racial and ethnic minority participants.

The 54 authors and co-authors of the 24 studies that included SMM participant data were largely women (72%): 39 women-identified authors to 15 men-identified authors. One author identified as a transgender man. Investigation of the authors’ disciplines revealed that 18 of the first or corresponding authors came from a psychological field while the remaining five were from other disciplines (i.e., Sociology; Social Work; Public Health; Criminology, Law, and Justice; Criminal Justice). Seven of the studies appear to be from the same research team (i.e., Dr. Gidycz and colleagues from Ohio University’s Department of Psychology).

## Discussion

This literature review and secondary data analysis focused on research exploring sexual violence perpetration by college men in the United States and Canada between 2000 and 2017. Despite this research team’s knowledge of existing sexual and gender minority (SGM) health disparities and heterosexism in sexual violence research, the lack of available data was still surprising. Of the 78 individual U.S college convenience samples representing the data of over 25,000 college men examined, none reported perpetration rates specifically among sexual minority men (SMM). The 24 articles that did report any SMM participant data were homogenous (e.g., over 90% heterosexual, White, cisgender participants).

No significant relationship was found between the inclusion of SMM participant data and measurement strategies designed to capture the unique experiences and needs of SGM populations. This is remarkable given that most studies did in fact use modified questionnaires; most researchers seem to have few compunctions with modifying standardized questionnaires like the Sexual Experiences Survey (SES). Yet, few made these modifications in the spirit of SGM inclusion. The majority of the studies including SMM data used a variation of the SES, consistent with the overall sample. The SES in its various original forms uses gendered language, particularly language equating genitalia to gender. By not using gender-neutral language, such questionnaires have the potential to alienate non-heterosexual and non-cisgender respondents as the reality of their lived experiences is not represented (Woodford & Kulick, 2015). Revising these questionnaires in an empirically-based manner to include gender-neutral language and experiences common among SGM people is important because it will allow researchers to better understand, and ultimately to represent, the reality of sexual violence among SGM individuals.

A significant relationship was found between studies reporting data from SMM and the date of study publication, with more recent studies (modal year 2017) reporting more comprehensive descriptions of their participants than studies from earlier years. This suggests a recent and rapid change coinciding with the publication of the 2016 APA’s “Resolution on Data About Sexual Orientation and Gender Identity” which strongly recommends that researchers report data regarding participants’ gender and sexual identity (American Psychological Association, 2016). This suggests the importance and quick impact of such policies.

The authors and co-authors of the 24 studies analyzed were mostly women (72%). One explanation for the predominance of women authors could be that historically, research on rape stemmed from feminist scholars and the feminist psychology movement (Rozee & Koss, 2001), which has been dominated by women.

There is a considerable health disparity among SGM groups; this is even more pronounced when stratifying by race. Lesbians of color report higher rates of sexual victimization when compared to other cultural groups (Descamps et al., 2000; Morris & Balsam, 2003). Unfortunately, we found that inclusion of SMM participant data in our analysis was not significantly associated with the inclusion of racial and ethnic minority data, hampering the ability to understand this complex health problem. the removal of sexual identity minority “outliers” from the overall sample, while this allows for more straightforward data analyses, excludes these vulnerable populations of interest.

The current gap in sexual perpetration literature within the LGBTQ+ community may also disproportionately exclude members of the same community who are committing acts of sexual violence (Potter et al., 2012). Robust collaboration and active engagement with SGM people should be a central research focus as we work towards shared goals: understanding the commonalities and differences in perpetration across ethnic, gender, and sexual minority people, decreasing vulnerability for sexual victimization in highly vulnerable groups, and developing new strategies for sexual violence prevention.

### Limitations

As documented in the original study (Anderson et al., 2021), this analysis examined the perpetration behavior of largely cisgender, college-attending males. We recognize that for a more granular characterization of sexual perpetration in the general college population, it is important to examine perpetration in various populations (e.g., cisgender women of color, trans men and women, and other gender non-conforming or non-binary people). However, even within the cisgender male population on college campuses, there is a significant lack of demographic data on whether these men were attracted solely to cisgender women or aligned with another sexuality that includes attraction to cisgender women (e.g., bisexual, pansexual, etc.). The included studies largely defined SGM people by sexual identity; yet research has shown the SM community would comprise 20% of women and 10% of men, compared to 6.4% and 3.6%, respectively, if same-sex attraction/behavior within the past year is included (Mishel, 2019). In addition to limitations in how SGM groups were defined, most of the questionnaires used do not account for perpetration tactics that may be specific to harming sexual minority people, such as threatening to “out'' the victim or other sexual identity-related hate crimes.

### Clinical Implications

The lack of available data on SMM in college samples presents serious clinical problems. Seeking mental health care is particularly difficult for SGM people. The dual stresses of being SGM in an often hostile culture and struggling with mental health issues presents a confounding alienation whereby some people feel isolated from groups of people aligned solely with either one of these two identities (Veltman & Chaimowitz, 2014). This is even more apparent for SGM people of color struggling with their mental health (Cyrus, 2017). Socioeconomic factors such as homelessness provide another reason for increased health disparities in the SGM community. For example, psychological disorders are particularly high among the homeless population (Keuroghlian et al., 2014) of which SGM people make up 20–40% (Ecker, 2016; Shelton & Abramovich 2019). Changes in our mental healthcare system are needed to equalize access to care (e.g., providing SGM-specific mental health educational materials). Collection and analysis of sexual minority data is paramount to clarifying and making transparent sexual minority-related health disparities and making relevant health care system changes (Wolff et al., 2017) that will specifically address the threat of sexual violence perpetration in sexual minority communities.

## Conclusion

Our research has illustrated a canyon-sized gap in the sexual perpetration literature regarding SGM people. Among samples of college men, SMM are under-sampled and left out of analyses if they are sampled at all. We recommend the field focus their efforts on conducting research that recognizes the complex sexual violence dynamics in SMM, an understudied and vulnerable group. Researchers should also use measures that include tactics specific to vulnerable groups and language that does not further marginalize gender and sexual minority people. Such changes are needed to make findings more generalizable, address the needs of marginalized groups of people, and ultimately, maximize our ability to decrease the public health threat of sexual perpetration.

## Data Availability

This article includes presentation of a secondary data analysis of a systematic review on college men's sexual perpetration rates and associated measurement strategies (Anderson et al., 2021).

## Appendix

**Table A1:** Subset of Studies From the Systematic Review (Anderson et al., 2021) Including Sexual Minority Men (N = 24)

Pub Year	Authors	Title	*N*	Publication	Pub Type	DOI
2016	Ambrose, Carrie; Gross, Alan	Interpreting Sexual Dating Encounters: Social Information Processing Differences in Men and Women	83 women; 94 men	Journal of Family Violence	journal article	https://doi.org/10.1007/s10896-015-9757-z
2017	Anderson, RaeAnn E.; Cahill, Shawn P.; Delahanty, Douglas L.	Initial Evidence for the Reliability and Validity of the Sexual Experiences Survey-Short Form Perpetration (SES-SFP) in College Men	402 men	Journal of Aggression, Maltreatment, & Trauma	journal article	https://doi.org/10.1080/10926771.2017.1330296
2012	Anthony, Elizabeth R.; Cook, Sarah L.	Assessing the impact of gender-neutral language on disclosure of sexual violence	258 women; 190 men	Psychology of Violence	journal article	http://dx.doi.org/10.1037/a0028562
2017	Barker, Analise; Galliher, Renee V.	A mediation model of sexual assault among Latter-Day Saints	131 women; 77 men	Journal of Aggression, Maltreatment & Trauma	journal article	https://doi.org/10.1080/10926771.2016.1272657
2016	Dardis, Christina M.; Murphy, Megan J.; Bill, Alexander C.; Gidycz, Christine A.	An investigation of the tenets of social norms theory as they relate to sexually aggressive attitudes and sexual assault perpetration: A comparison of men and their friends	200 men	Psychology of Violence	journal article	http://dx.doi.org/10.1037/a0039443
2016	Elias-Lambert, Nada; Black, Beverly M.	Bystander Sexual Violence Prevention Program: Outcomes for High- and Low-Risk University Men	142 men	Journal of Interpersonal Violence	journal article	https://doi.org/10.1177/0886260515584346
2011	Gidycz, Christine A.; Orchowski, Lindsay M.; Berkowitz, Alan D.	Preventing Sexual Aggression Among College Men: An Evaluation of a Social Norms and Bystander Intervention Program	635 men	Violence Against Women	journal article	https://doi.org/10.1177/1077801211409727
2007	Gidycz, Christine A.; Warkentin, Jennifer B.; Orchowski, Lindsay M.	Predictors of perpetration of verbal, physical, and sexual violence: A prospective analysis of college men	425 men	Psychology of Men & Masculinity	journal article	https://doi.org/10.1037/1524-9220.8.2.79
2017	Holmgreen, Lucie; Oswald, Debra L.^a^	Men’s Sexual Coerciveness, Perceptions of Women’s Attachment, and Dating Preferences	130 men	Violence and Victims	journal article	https://doi.org/10.1891/0886-6708.VV-D-12-00133
2017	Johnson, Shannon M.; Murphy, Megan J.; Gidycz, Christine A.	Reliability and Validity of the Sexual Experiences Survey–Short Forms Victimization and Perpetration	433 women; 136 men	Violence and Victims	journal article	https://doi.org/10.1891/0886-6708.VV-D-15-00110
2004	Johnson, Thomas J; Stahl, Courtney	Sexual Experiences Associated with Participation in Drinking Games	309 women; 181 men	The Journal of General Psychology	journal article	N/A
2005	Loh, Catherine; Gidycz, Christine A.; Lobo, Tracy R.; Luthra, Rohini	A Prospective Analysis of Sexual Assault Perpetration: Risk Factors Related to Perpetrator Characteristics	325 men	Journal of Interpersonal Violence	journal article	https://doi.org/10.1177/0886260505278528
2002	Marmelstein Blackwell, Lisa	Sexual aggression among college men: A test of two empirical models	293 men	State University of New York at Binghamton	thesis	N/A
2003	Ménard, Kim S.; Hall, Gordon C. Nagayama; Phung, Amber H.; Ghebrial, Marian F. Erian; Martin, Lynette	Gender Differences in Sexual Harassment and Coercion in College Students: Developmental, Individual, and Situational Determinants	278 women; 148 men	Journal of Interpersonal Violence	journal article	https://doi.org/10.1177/0886260503256654
2016	Murphy Austin, Megan J.; Dardis, Christina M.; Wilson, Milo S.; Gidycz, Christine A.; Berkowitz, Alan D.	Predictors of Sexual Assault–Specific Prosocial Bystander Behavior and Intentions: A Prospective Analysis	273 men	Violence Against Women	journal article	https://doi.org/10.1177/1077801215597790
2016	Pickett, Scott M.; Parkhill, Michele R.; Kirwan, Mitchell	The influence of sexual aggression perpetration history and emotion regulation on men’s aggressive responding following social stress.	135 men	Psychology of Men & Masculinity	journal article	https://doi.org/10.1037/men0000032
2009	Schewe, Paul A.; Adam, Najma M.; Ryan, Kathryn M.	A Qualitative Analysis of the Temptation to Use Force in Sexual Relationships	83 men	Violence and Victims	journal article	https://doi.org/10.1891/0886-6708.24.2.219
2009	Stephens, Kari A.; George, William H.	Rape Prevention With College Men: Evaluating Risk Status	146 men	Journal of Interpersonal Violence	journal article	https://doi.org/10.1177/0886260508319366
2008	Thompson, Edward H.; Cracco, Elizabeth J.	Sexual Aggression in Bars: What College Men Can Normalize	264 men	The Journal of Men's Studies	journal article	https://doi.org/10.3149/jms.1601.82
2017	Tuliao, Antover P.; McChargue, Dennis E.; Klanecky, Alicia K.	Information acquisition in sexual aggression.	167 men	Psychology of Violence	journal article	https://doi.org/10.1037/vio0000157
2010	Voller, Emily K.; Long, Patricia J.	Sexual Assault and Rape Perpetration by College Men: The Role of the Big Five Personality Traits	521 men	Journal of Interpersonal Violence	journal article	https://doi.org/10.1177/0886260509334390
2007	Warkentin, Jennifer B.; Gidycz, Christine A.	The Use and Acceptance of Sexually Aggressive Tactics in College Men	297 men	Journal of Interpersonal Violence	journal article	https://doi.org/10.1177/0886260507301793
2010	Widman, Laura; McNulty, James K.	Sexual Narcissism and the Perpetration of Sexual Aggression	147 women; 152 men	Archives of Sexual Behavior	journal article	https://doi.org/10.1007/s10508-008-9461-7
2017	Young, Belinda-Rose; Desmarais, Sarah L.; Baldwin, Julie A.; Chandler, Rasheeta	Sexual Coercion Practices Among Undergraduate Male Recreational Athletes, Intercollegiate Athletes, and Non-Athletes	379 men	Violence Against Women	journal article	https://doi.org/10.1177/1077801216651339

^a^Holmgreen and Oswald (2017) included SMM data but excluded from analysis any participants that indicated they were "completely homosexual" as ascertained through self-report on a continuum scale for sexual orientation. Therefore, SMM data was included in their analysis only for participants who reported falling somewhere between "completely heterosexual" and "completely homosexual."
